# Extracellular vesicles from mesenchymal stromal cells primed with synthetic toll-like receptor 4 agonists treat hematopoietic acute radiation syndrome

**DOI:** 10.1093/stcltm/szaf068

**Published:** 2026-01-09

**Authors:** John A Kink, Matthew H Forsberg, Derek M Krismer, Anna S Thickens, Raghavan Chinnadurai, Alex S Chen, Daniel J Chacko, Melissa Graham, Peiman Hematti, Christian M Capitini

**Affiliations:** Department of Medicine, University of Wisconsin School of Medicine and Public Health, Madison, WI 53705, United States; University of Wisconsin Carbone Cancer Center, Madison, WI 53705, United States; Department of Pediatrics, University of Wisconsin School of Medicine and Public Health, Madison, WI 53705, United States; Department of Pediatrics, University of Wisconsin School of Medicine and Public Health, Madison, WI 53705, United States; Department of Medicine, University of Wisconsin School of Medicine and Public Health, Madison, WI 53705, United States; Department of Medicine, University of Wisconsin School of Medicine and Public Health, Madison, WI 53705, United States; Department of Biomedical Sciences, Mercer University School of Medicine, Savannah, GA 31404, United States; Department of Medicine, University of Wisconsin School of Medicine and Public Health, Madison, WI 53705, United States; Department of Pediatrics, University of Wisconsin School of Medicine and Public Health, Madison, WI 53705, United States; Department of Medicine, University of Wisconsin School of Medicine and Public Health, Madison, WI 53705, United States; The Comparative Pathology Laboratory, Research Animal Resource Center, University of Wisconsin, Madison, WI 53705, United States; Division of Hematology/Oncology, Medical College of Wisconsin, Milwaukee, WI 53226, United States; University of Wisconsin Carbone Cancer Center, Madison, WI 53705, United States; Department of Pediatrics, University of Wisconsin School of Medicine and Public Health, Madison, WI 53705, United States

**Keywords:** extracellular vesicles, exosomes, mesenchymal stromal cells, TLR4, hematopoietic acute radiation syndrome, monocytes, radiomitigator

## Abstract

Whole-body exposure to ionizing radiation can lead to cellular DNA damage to bone marrow (BM), causing lethal hematopoietic acute radiation syndrome (H-ARS). Extracellular vesicles (EVs) from human BM-derived mesenchymal stromal cells were primed with CRX-527 (CRX), a synthetic TLR4 agonist, characterized and tested as a radiomitigator therapy. Using a xenogeneic H-ARS mouse model, a single *in vivo* treatment with CRX-EVs administered 4 or 24 hours after lethal irradiation significantly improved weight loss, clinical scores and prolonged survival compared to control treatments. *Ex vivo* generation of CRX-EV educated monocytes (CRX-EEMos) were also effective in a H-ARS model when administered 24 hours after lethal irradiation. CRX-EVs or CRX-EEMos significantly promoted hematopoiesis in BM and potentially the spleen, leading to restoration of peripheral complete blood counts. CRX-EEMos showed increased gene expression of IL-6 and IL-10: enriched for PD-L1 but low for CD16 in CD14-expressing monocytes. Antisense inhibition of Let-7 microRNAs in CRX-EEMos suppressed IL-10 gene expression and protein secretion, implicating a novel role for Let-7 in radioprotection. CRX-EVs can effectively treat H-ARS by increasing the secretion of anti-inflammatory molecules while stimulating monocytes to promote hematopoiesis in BM. The potential for large-scale production of CRX-EVs as an “off-the-shelf” treatment for H-ARS makes them a potential medical countermeasure for radiological and nuclear threats.

## Graphical abstract

**Graphical Abstract:**
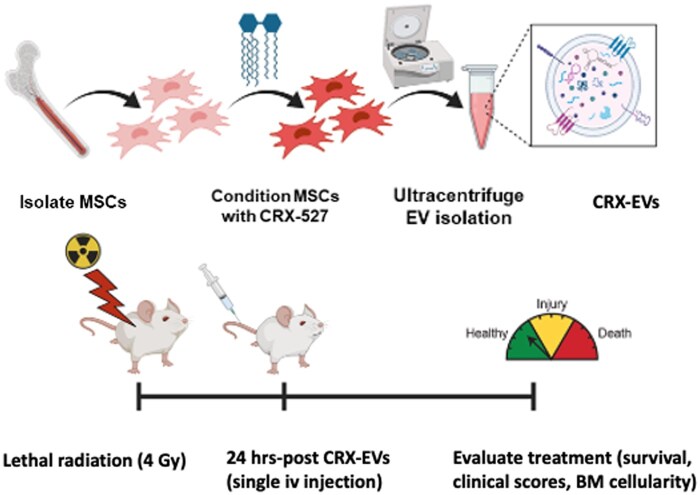

Significance statement Exposure to lethal doses of ionizing radiation can cause acute radiation syndrome (ARS) that can affect hematopoietic cells within the bone marrow (H-ARS). Potential exposure from a radiopharmaceutical therapy or nuclear accidents increases the need for effective radiation countermeasures as a national priority. A single treatment of extracellular vesicles (EVs) from mesenchymal stromal cells primed with a synthetic toll-like receptor-4 agonist (CRX) effectively prevents H-ARS in mice when administered 4 or 24 hours after lethal irradiation. CRX-EVs educate monocytes into a radioprotective phenotype which secretes anti-inflammatory molecules like IL-10, regulated in part by Let-7 microRNAs, and growth factors that promote immune modulation and hematopoietic recovery. CRX-EVs can be produced in large quantities and cryopreserved for immediate use in case of mass casualty events after a radiation event. Future studies involving the determination of the molecular mechanisms of action and pharmacokinetics in immunocompetent large animals will inform first-in-human clinical trials with CRX-EVs.

## Introduction

Protecting the health of military personnel, emergency response teams, and healthcare workers from the effects of radiological or nuclear hazards is essential to national defense.^1-4^ Radiation exposure after the detonation of a tactical or low-yield nuclear weapon during war or a terrorist attack, recently thought of as unlikely, may be more than just a hypothetical scenario.^5^ Danger to the population from accidents at nuclear power plants or from high radiation exposure to astronauts in space are also sources of concern.^6^^,^^7^ Exposures to ionizing radiation in all of these circumstances can lead to multi-system health effects collectively known as acute radiation syndrome (ARS).^8^ Since radiation effects actively dividing cells, hematopoietic stem cells in the bone marrow (BM) are most vulnerable, causing hematopoietic-ARS (H-ARS).^9^ Even low radiation dose exposures of 1-2 Gray (Gy) can result in moderate leukopenia while higher doses of 2-6 Gy can result in various degrees of pancytopenia, causing life-threating infections, anemia and hemorrhage with mortalities from 5% to 50%, even with medical support.^10^^,^^11^

Therapeutic countermeasures available to date focus on hematopoietic growth factors and/or the replacement of blood cells. Indeed, the current Food and Drug Administration (FDA)-approved therapies to treat H-ARS, filgrastim and pegfilgrastim, are both granulocyte colony stimulating factors which promote the production of neutrophils by the BM.^12^ However, these treatments surprisingly have yet to show a statistical decrease in mortality in radiation-injured humans.^13^ Allogeneic hematopoietic stem cell transplant is also an option, but is limited by the care coordination needed to quickly mobilize hematopoietic stem cells from donors. During this time patients with H-ARS are at the highest risk of death, and finding volunteer donors to treat patients in a mass-exposure event would prove to be very difficult. Consequently, there is an urgent unmet medical need to develop a more practical, off-the-shelf, effective countermeasure to treat H-ARS.^14^

Extracellular vesicles (EVs) are small lipid bilayer particles released by cells which play a key role in intercellular communication through the transfer of bioactive cargo components.^15^ These components largely consist of proteins and nucleic acids such as micro-RNA (miRNA) that control gene expression in the recipient cell.^16^ BM-derived mesenchymal stromal cells (BM-MSCs) are multipotent cells involved in hematopoietic support, tissue repair and immune regulation.^17^ Importantly, EVs released from BM-MSCs are key mediators that transmit these homeostatic functions to effector cells involved in these downstream processes.^18^ Previously we showed that EVs from BM-MSCs could educate macrophages or monocytes *ex vivo* into an unique anti-inflammatory and regenerative M2-like phenotype.^19^^,^^20^ Moreover, BM-MSCs primed with *E. coli* lipopolysaccharide (LPS), a Toll-like receptor-4 (TLR-4) agonist, generated LPS-EVs which polarized macrophages or monocytes into an effective radio-protective M2-like phenotype that prolonged survival by stimulating hematopoiesis in the BM and complete blood counts (CBCs) recovery in a H-ARS model.^19^^,^^20^ Furthermore, the direct treatment with LPS-EVs alone was shown to be effective in a H-ARS model. In that report, we also demonstrated the feasibility of producing large-scale amounts of EVs for a mass exposure event from MSCs using a hollow-fiber bioreactor system.^21^

A limitation of this approach involves the use of LPS, which if carried over in EV production may result in a septic-like presentation in already immunocompromised H-ARS patients. Therefore, replacement with a safer, more clinically suitable TLR-4 priming agent is needed. CRX-527 (CRX), a TLR-4 mimetic with a synthetic lipid A tail without endotoxin displays similar responses in human monocytes to LPS by transcriptional profiling.^22^ Here, we compare yields and physical aspects including surface marker profiles between CRX-EVs and unprimed MSC-EVs. We tested the efficacy of CRX-EVs directly, and of CRX-EV-educated monocytes (CRX-EEMos), in a xenogeneic H-ARS mouse model. CRX-EEMos were characterized for changes in gene expression, secretome, and cell surface marker profile. To understand how mice respond to human CRX-EVs in the H-ARS model, gene expression analysis of murine monocytes was conducted. Antisense oligonucleotides against Let-7 miRNAs enriched in CRX-EVs were conducted to determine which cytokines are regulated during radioprotection.^23^ Overall, these results indicated that the direct treatment of CRX-EVs was effective against lethal radiation *in vivo* by polarizing monocytes/macrophages into an anti-inflammatory and regenerative phenotype and has the potential to be a safe and effective “off the shelf” countermeasure to treat H-ARS.

## Materials and methods

### Isolation and characterization of EVs from human MSCs

Human BM-MSCs were isolated from multiple healthy donors using an institutional review board (IRB)-approved protocol at the University of Wisconsin-Madison (2016-0298).^24^ Identity of BM-MSCs was confirmed by flow cytometry as previously described.^24^ BM-MSCs were grown in T75 cm^2^ filter cap cell culture flasks (Greiner Bio-One, Monroe, NC, USA) as described in Supplementary methods.

To isolate MSC-EVs or CRX-EVs, early passage BM-MSCs were expanded to confluence, (approximately 10^6^ cells per flask) in T-75 flasks, washed with phosphate-buffered saline (PBS) (Hyclone, Logan, UT, USA), and replaced with MSC serum-free media (SFM) (StemPro A103332-01, ThermoFisher Scientific, Waltham, MA, USA). To produce CRX-EVs, the same BM-MSC isolate was primed with 0.1 µg/mL CRX-527 (InvivoGen, Vista Sorrento Pkwy, San Diego, CA, USA) in SFM. MSC-EVs and CRX-EVs were isolated using a centrifugation process (Supplementary methods) as described previously.^25^ The EVs were quantitated for protein and RNA concentration using a NanoDrop spectrophotometer (Thermo-Fisher, Waltham, MA, USA). Particle size and concentration were quantitated using a Zetaview (Particle Metrix Inc, Mebane, NC, USA) or a Nanosight NS300 (Nanosight LTD, Malvern PA) by Zen-Bio Inc, (Durham, NC, USA). Residual LPS (endotoxin) and surrogate assay for residual CRX-527 was determined using a chromogenic Limulus Amebocyte Assay (LAL) performed by VRL/Eurofins (Centennial, CO, USA) using EVs prepared from LPS-stimulated MSC’s as positive controls.^19^

### Surface marker and Western blot analysis of EVs

Surface marker characterization of MSC-EVs or CRX-EVs from two different human MSC isolates was performed by flow cytometry using the MACSPlex EV Kit. (Miltenyi Biotec, Bergisch Gladbach, Germany) This kit detects 37 known EV surface markers: CD105, CD11c, CD133/1, CD14, CD142, CD146, CD19, CD1c, CD2, CD20, CD209, CD24, CD25, CD29, CD3, CD31, CD326, CD4, CD40, CD41b, CD42a, CD44, CD45, CD49e, CD56, CD62P, CD63, CD69, CD8, CD81, CD86, CD9, HLA-ABC, HLA-DRDPDQ, ROR1, MCSP, and SSEA-4. Analysis was performed as described (Supplementary methods) with values of 1.0 or more considered as positive as described.^20^ The detection of heat shock protein 70 (Hsp70), a known cytosolic protein marker in MSC-EVs was also performed by Western blot as described in the Supplementary methods.

### Isolation and education of monocytes by EVs

Human monocytes were isolated from healthy donor peripheral blood using an institutional review board (IRB)-approved protocol (2016-0298). CD14^+^ monocytes were isolated using anti-human CD14 microbeads (Miltenyi Biotec, Bergisch Gladbach, Germany) and an autoMACS Pro Separator instrument (Miltenyi Biotec) as directed by the manufacturer.^24^ Monocytes were cultivated in culture medium and educated with the same concentration of MSC-EVs, CRX-EVs, or uneducated (controls) for 18-24 hours as described in Supplementary methods.^20^

### H-ARS mouse model

Age matched (8-16 weeks) male and female NOD scid gamma (NSG) mice [NOD.Cg-*Prkdc^scid^ Il2rg^tm1Wjl^*/SzJ (NSG)] were used (The Jackson Laboratory, Bar Harbor, Maine, USA). The Animal Care and Use Committee at the University of Wisconsin-Madison approved all experimental animal protocols (M005915). On day 0, NSG mice received a lethal 4 Gy whole-body radiation using an X-RAD 320 kV irradiator (Precision X-Ray, North Branford, CT, USA) or a CIX3 320 kV irradiator (Xstrahl, Inc, Suwanee, GA, USA). For direct treatment studies, PBS (Hyclone) (controls), 5 × 10^9^ MSC-EVs or CRX-EVs, in 100 µl PBS was administered intravenously (i.v.) by tail vein at 4 or 24 hours after radiation challenge. The 4-hour time selection served as an early time point, while the 24-hour time point was based on NIH guidance as a reasonable time-frame for a countermeasure treatment that would mimic a clinical scenario. For cell treatment studies, 10^7^ cells of uneducated control monocytes, EEMos, or CRX-EEMos in 100 µl PBS were administered once i.v. by tail vein 24 hours after radiation challenge. To assess the effect of direct treatment of mice with CRX-527 in the ARS model, NSG mice were each treated with 1 µg of CRX-527 in PBS by tail vein 4 hours after radiation with 4 Gy. The dose selected was based if the entire amount of CRX-527 was carried over in the given EV dose/mouse. Mice were monitored for survival and clinical signs of ARS using a clinical scoring system (Supplementary methods) as previously described.^26^ Mice suspected of contracting *Corynebacterium bovis* infection were confirmed by qPCR using services provided by Charles River Research Animal Diagnostic Services. (Wilmington, MA, USA) and treated with enrofloxacin (Baytril, Elanco Animal Health, Greenfield, IN, USA). CBCs were performed to determine the effects of radiation by comparing pre-radiation with postradiation blood in mice.

### Diagnostic necropsy and histologic preparation

Histology was performed on BM from the long bones (femur) of nonirradiated mice or mice in the H-ARS model as described in Supplementary methods from two independent studies and collected at various days postradiation (day 7, 27, 49, and 133). Cellularity loss in the BM was evaluated in a blinded manner by M.G. using a semi-quantitative scoring system based on guidance for nonclinical toxicity studies.^27^ Images were presented at 20x power to best demonstrate the cellularity scores made by the histologist. Splenic weights were taken directly or presented as a percentage of spleen weight to total body weight (% BW) to assess for extramedullary hematopoiesis.

### Gene expression analysis of EV-educated human or mouse monocytes

Human primary monocytes were isolated from 3 to 4 donors and educated with 5 × 10^9^ MSC-EVs or CRX-EVs per 10^7^ cells and were seeded into T75 culture flasks in monocyte culture medium. RNA was purified from uneducated control and educated monocytes using a RNeasy micro kit, (Qiagen, Valencia, CA, USA) converted to cDNA using Verso cDNA synthesis kit (Thermo Scientific, Pittsburgh, PA, USA) and quantitative polymerase chain reaction (qPCR) was performed and analyzed as described in Supplementary methods. Verified human primers (Qiagen) used for gene expression included IL-6, IL-7, IL-8, IL-10, IL-13, indoleamine 2,3-dioxygenase (IDO), fibroblast growth factor 2 (FGF2), chemokine ligand 5 (CCL-5), tumor necrosis factor alpha (TNFα), macrophage inflammatory protein 1a (MIP-1α), macrophage inflammatory protein 1b (MIP-1β), granulocyte-macrophage colony-stimulating factor (GM-CSF) and granulocyte-colony stimulating factor (G-CSF).

For measuring murine monocyte gene expression, primary monocytes from C57BL/6 mice (Cellero, Northridge, CA, USA) at 10^7^ cells were educated with 5 × 10^9^ human MSC-EVs or CRX-EVs in DMEM with 10% FBS. Isolation of RNA, cDNA and qPCR analysis were performed as described above except using verified mouse primers (Qiagen) for IL-6, IL-10, IL-13, IDO, CCL-5, GM-CSF, and G-CSF.

### Flow cytometry of EV-educated human monocytes

Monocytes from 3 to 4 healthy human donors were generated as controls (uneducated), EEMos and CRX-EEMos, counted with a Z1 Particle Counter (Beckman Coulter, Brea, CA, USA) and then 1 × 10^6^ cells were incubated with Fc block (BD Pharmingen, San Jose, CA, USA) for 10 min at room temperature. Cells were then stained at 4 °C for 20-30 min with anti-human antibodies in staining buffer (PBS with 2% FBS). All antibodies were purchased from BioLegend (San Diego, CA, USA) and included: CD206: (15-2, cat# 321105), CD163: (GHI/61, cat# 333617), PD-L1: (29E.2A3, cat# 329721), PD-L2: (24F.10C12, cat# 329608), CD14: (HCD14, cat# 325627), CD16: (3G8, cat# 302025), HLA-DR: (L243, cat# 307639), and CD86: (IT2.2, cat# 305431). Flow cytometric analysis of monocytes was performed as described in Supplementary methods.

### Secretome profiling of EV-educated monocytes

Monocytes isolated from three healthy human donors were placed into 48 well plates (approximately 2 × 10^5^ cells/well) in 250 µL of culture media as either uneducated monocytes (controls) or educated with 1 × 10^8^ of MSC-EVs or CRX-EVs to generate EEMos and CRX-EEMos, respectively. A second independent experiment was performed using the same three donors, except education using a different set of production lots of MSC-EV and CRX-EVs. After education for 18-24 hours, the culture media of the EEMos and CRX-EEMos were collected and centrifuged at 300 × *g* for 10 min to pellet debris and supernatant was assayed for secreted factors. Assay controls included culture media alone and culture media spiked with MSC-EVs or CRX-EVs. Thirty secreted factors were analyzed using the Cytokine Human Magnetic 30-Plex Panel for the Luminex™ platform (LHC6003M, ThermoFisher Scientific) according to manufacturer’s instructions. Each supernatant was assayed in triplicate for the following analytes: IL-1RA, IL-1β, IL-2, IL-2R, IL-4, IL-5, IL-6, IL-7, IL-8, IL-10, IL-12 (p40/p70), IL-13, IL-15, IL-17, interferon alpha (IFNα), interferon-gamma (IFNγ), interferon gamma-induced protein 10 (IP-10), monokine induced by IFNγ (MIG)/CXCL9, epidermal growth factor (EGF); hepatocyte growth factor (HGF), vascular endothelial growth factor A (VEGF), chemokine ligand 5 (CCL-5)/regulated upon activation normal T cell expressed and secreted (RANTES), eotaxin, TNFα, fibroblast growth factor 2 (FGF-basic), monocyte chemoattractant protein 1 (MCP-1), MIP-1α/CCL-3, MIP-1β/CCL-4, GM-CSF, and G-CSF. Analytes in the supernatants were detectable at the pg/ml range using the Luminex xMAP platform.

### Let-7 miRNA inhibition studies in EV-educated monocytes

Primary human control monocytes, EEMos and CRX-EEMos were either uninhibited or treated with miRNA antisense inhibitors to Let-7b/Let-7d or a nonsense control and compared for changes in gene expression by qPCR and protein secretion by multiplex ELISA as described in Supplementary methods.

### Statistical analysis

Statistics were performed using Microsoft Excel and GraphPad Prism version 8.1. (GraphPad Software, San Diego, CA, USA) Data was reported as mean ± standard deviation (SD) or standard error of the mean (SEM) as noted. Pairs of data were compared using multiple *t*-tests while groups of 3 or more were compared using ordinary one-way ANOVA or Kruskal–Wallis test with Dunn’s post-test comparison. Mantel–Cox log-rank was used for the comparison of Kaplan–Meier survival curves. Principal component analysis and *t* tests were performed to compare pairs of CBC data. A *P* value less than 0.05 was considered statistically significant for all tests.

## Results

### CRX-EVs showed an elevated surface marker for CD44 compared to control EVs

**Figure 1.**
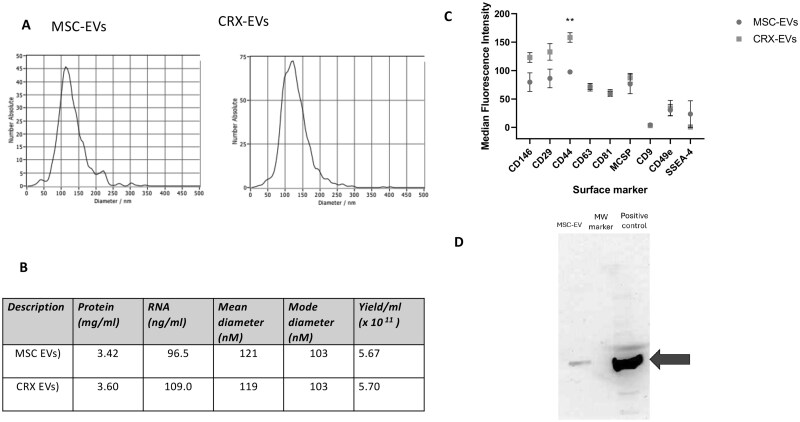
Comparison of EVs and CRX-EVs isolated from human MSCs. EVs from unprimed human MSCs (MSC-EVs) or MSCs primed with CRX (CRX-EVs) were analyzed for (A) size distribution range characterized by dynamic light scattering (B) protein, RNA content, particle sized distributions (mean and mode), and yields (C) cell surface markers and (D) cytosolic marker Hsp70 by Western blot. The EVs were stained with 37 different EV antibody surface markers and the mean fluorescence intensity were compared relative to isotype controls from two independent studies. ^**^P ≤ .01.

We found that several properties of EVs generated from CRX-primed MSCs were like EVs generated from unprimed MSCs. Dynamic light scattering analysis showed a similar number and diameter comparing a representative set of MSC-EVs and CRX-EVs from one isolate (Figure 1A). When comparing EVs from matched set preparations from three different MSC isolates, the mean and mode diameters for MSC-EVs and CRX-EVs were 121 nm ± 12 and 119 nm ± 15, and the modes were 103 nm ± 18 and 103 ± 19, (*P *> .5), respectively (Figure 1B). Total protein content for MSC-EVs and CRX-EVs were similar at 3.42 mg/mL ± 0.47 and 3.6 mg/mL ± 0.54, whereas total RNA content was 96.5 ng/mL ± 26.4 and 109.2 ± 9.2, (*P *> .5), respectively (Figure 1B). The mean particle yields based on conditioned media volume (300 mL) from both groups were also similar; MSC-EVs at 5.67 × 10^11^ particles/mL and CRX-EVs at 5.7 × 10^11^ particles (*P *> .5). The LAL assay used to detect residual CRX in the EVs were below the limit of detection at 0.05 EU/ml for CRX-EVs (data not shown). Analysis of the cell surface marker profile of both MSC-EVs and CRX-EVs showed a common core set of surface markers, each possessing 9 out of a total of 37 surface markers examined, with CRX-EVs showing a significantly elevated level of surface marker for CD44 compared to MSC-EVs (Figure 1C). Hsp70 widely recognized as a cytoplasmic EV cargo protein was also present by Western blot in isolated MSC-EVs (Figure 1D).

### CRX-EVs administered 4- or 24-hours postradiation protected mice in a H-ARS model

**Figure 2.**
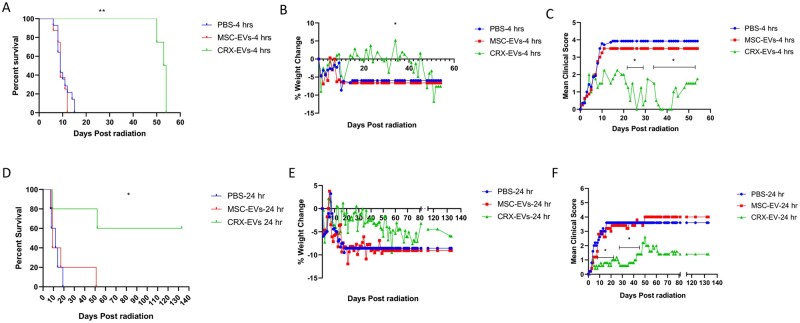
CRX-EVs administered 4 hours or 24 hours after lethal radiation are effective against H-ARS in xenogeneic mouse models. On day 0, NSG mice received 4 Gy of lethal radiation followed by an i.v. treatment administered 4 hours later **(**A-C) or 24 hours later (D-F) with vehicle (PBS), 5 × 10^9^ EVs from unprimed MSCs (MSC-EVs) or EVs from CRX-primed MSCs (CRX-EVs). (A) Survival curve of irradiated mice treated 4 hours after radiation. ^**^P ≤ .005 in mice treated with CRX-EVs compared to mice treated with PBS or MSC-EVs. (B) Mean % weight ^*^P ≤ .05 at day 34 in CRX-EV treated mice compared to mice treated with either PBS or MSC-EVs. (C) Mean clinical score (collective score of percent weight loss, posture, activity, and fur texture) ^*^P ≤ .05 between day 21 through 53 in CRX-EV treated mice versus treatments with PBS or MSC-EVs. (D) Survival curve of irradiated mice treated 24 hours after radiation. ^*^P ≤ .05 in mice treated with CRX-EVs compared to mice treated with PBS or MSC-EVs. (E) Mean % weight. (F) Mean clinical score ^*^P ≤ .05 day 9 to 23 and day 26 to 46 in CRX-EV treated mice versus treatments with PBS or MSC-EVs. The final mean percent weight change and mean clinical score were carried over after death to allow for statistical comparisons between groups. Log-rank (Mantel–Cox) was used to compare survival curves and Kruskal–Wallis with Dunn’s post-test was used for comparing % weight change and mean clinical scores. Results of A-C are pooled from three independent experiments, with 8 to 14 mice/group and D-F had 5 mice/group.

Efficacy of a single treatment of CRX-EVs was compared to vehicle (PBS) and MSC-EVs administered after lethal irradiation in a xenogeneic H-ARS mouse model. Long-term survival was significantly prolonged when CRX-EVs were infused 4 hours after lethal irradiation (Figure 2A). Median survival after radiation challenge for both PBS and MSC-EV treated mice was 9 days compared to 54 days in the CRX-EV treated group. Weight loss was significantly attenuated after CRX-EV treatment on day 34 (Figure 2B). CRX-EV treatment also significantly improved mean clinical scores from day 21 to 53, compared to PBS-controls; while a subset within the same span of time (day 23-26, 29-30, and 35-42) were also significantly improved compared to MSC-EV treated mice (Figure 2C). The protective effect of a single dose of CRX-EVs began to wane at day 45-50; during which both clinical scores and % weight change began to worsen until all the mice died by day 54.^19^

Since most radio-mitigator treatments would unlikely be administered only 4 hours postradiation exposure, a single infusion of CRX-EVs was given 24 hours after lethal irradiation in the xenogeneic H-ARS mouse model. CRX-EVs significantly prolonged survival in this setting (Figure 2D) with a duration of response to about day 50. This late-stage relapse appeared to be due to a secondary infection rather than from the classic toxicity from H-ARS as PCR analysis of skin swabs confirmed *Corynebacterium bovis*.^28^ After a single 1-week course of enrofloxacin (Baytril) on day 63, mice rapidly recovered and remained healthy throughout the length of the study. Median survival for PBS and MSC-EV treated mice was 12 days and 9 days, respectively, while CRX-EV treated mice remained healthy until day 133, when the study was terminated for histopathologic analysis of BM. Weight loss improved, notably on day 17 (Figure 2E), and clinical scores significantly improved between day 8-24 and 27-47 (Figure 2F) in mice treated with CRX-EVs. In contrast with these results using EVs from CRX-527 stimulated MSCs, the direct treatment of mice with CRX-527 administered 4 hours after lethal radiation did not significantly improve survival or lessen the severity of clinical scores in the mice (Figure S1, see online supplementary material for a color version of this figure).

Hematologic recovery was also observed when CRX-EV treatment was given 24 hours-post-irradiation (Table S1). Early postradiation (day 7), CBCs showed significant pancytopenia for all treatment groups compared to pre-radiation controls. By day 20, CRX-EV treated mice showed recovery of RBCs, WBCs, and lymphocytes to numbers comparable to pre-radiation levels. In contrast, by day 19-20, the single survivors of lethal irradiation after PBS or MSC-EV treatments continued to show pancytopenia. The CBC of a single MSC-EV treated mouse improved somewhat at day 43, but the mouse died shortly thereafter, while CBCs from CRX-EV treated mice remained normal up to day 75.

### CRX EEMos administered 24 hours postradiation protected mice from lethal ARS

**Figure 3.**
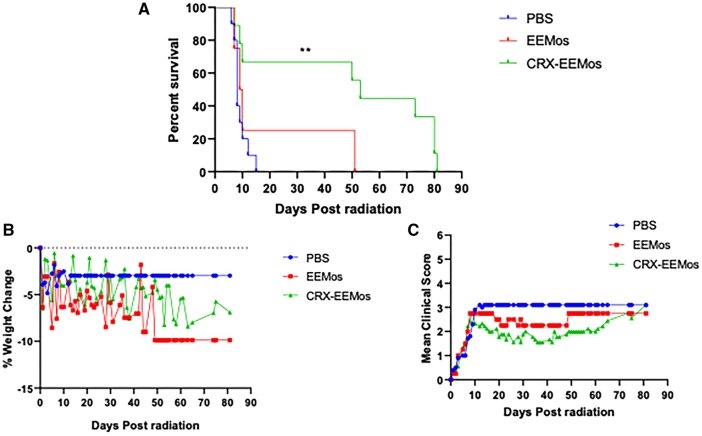
CRX-EEMos administered 24 hours after lethal radiation significantly improves survival from H-ARS in xenogeneic mouse models. On day 0, NSG mice received 4 Gy of lethal radiation followed by an i.v. treatment administered 24 hours later with vehicle (PBS), 1× 10^7^ monocytes educated with unprimed MSC-EVs (EEMos), or 1 × 10^7^ monocytes educated with EVs from CRX-primed MSCs (CRX-EEMos). (A) Survival curve of treated mice after radiation. ^**^P ≤ .005 in mice treated with CRX-EEMos compared to controls. (B) Mean percent weight change. (C) Mean clinical scores (percent weight loss, posture, activity, and fur texture). For B and C, the final mean % weight change and mean clinical score were carried over after death to allow for statistical comparisons between groups. Log-rank (Mantel–Cox) was used to compare survival curves and Kruskal–Wallis with Dunn’s post-test was used for comparing % weight change and mean clinical scores. Results were pooled from three independent experiments with 4 to 10 mice/group.

After establishing the protective effect of CRX-EVs 4 or 24 hours post-lethal irradiation due to improved hematopoietic recovery, we next determined if *ex vivo* education of human monocytes with EVs from CRX-primed human MSCs (CRX-EEMos) could achieve a similar effect. A single treatment of CRX-EEMos administered 24 hours-postradiation was also effective in prolonging the survival of mice with H-ARS as compared to mice treated with vehicle (PBS) or monocytes educated with unprimed MSC-EVs (EEMos). Median survival for the PBS-treated or EEMos were similar at 8 and 9.5 days, respectively, compared to 53 days using CRX-EEMos (Figure 3A). Notably, half of CRX-EEMo treated mice survived beyond day 70. No statistical improvement in either weight loss (Figure 3B) or clinical scores (Figure 3C) were present (Figure 2E and F). However, restoration of peripheral CBCs to pre-radiation values were observed in mice treated with CRX-EEMos (Table S2). While there was a steep initial induction of pancytopenia in all treatment groups early postradiation challenge (day 5 and 6), WBCs, neutrophils, lymphocytes and monocytes from CRX-EEMos treated mice were restored to pre-radiation levels by day 29 and 30.

### Both CRX-EVs and CRX-EEMos protect hematopoietic tissue in the bone marrow after H-ARS

**Figure 4.**
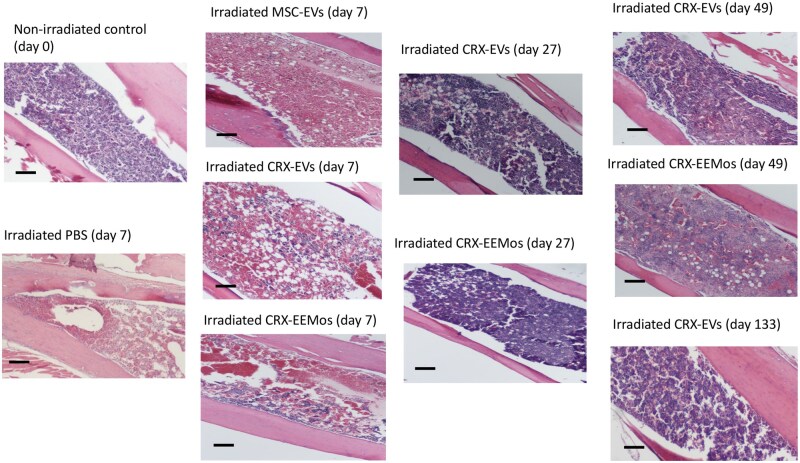
Human CRX-EVs or CRX-EEMos protect BM from tissue damage after lethal radiation. NSG mice received either no radiation (nonirradiated control) or were irradiated with 4 Gy followed by a single i.v. treatment 4 hours later with vehicle (PBS), 5 × 10^9^ MSC-EVs, 5 × 10^9^ CRX-EVs, or 1 × 10^7^ CRX-EEMos. A representative histology slide of BM from femurs is shown from healthy nonirradiated controls (day 0), PBS, MSC-EVs, CRX-EVs, or CRX-EEMos (day 7 postradiation), CRX-EVs or CRX-EEMos (day 27 postradiation), and CRX-EVs or CRX-EEMos (day 49 postradiation). Long-term surviving mice treated with CRX-EVs 24 hours after radiation also maintained BM recovery at day 133. Representative slides are 20x images of hematoxylin and eosin (H&E)-stained femoral BM sections from 3 to 4 mice per group. Scale bar = 100 microns.

To determine if CBC recovery correlated with the presence of hematopoietic cells within the BM, histopathological analysis of the BM from long bones at different times post-radiation of mice treated with CRX-EVs or CRX-EEMos were compared to nonirradiated control mice, PBS treated mice or mice treated MSC-EVs. Representative slides from 3 to 4 different mice per group at different time points postradiation were analyzed. Compared to nonirradiated controls, at 7 days post-irradiation, both PBS and MSC-EV treated mice showed almost a complete absence of hematopoietic cellularity (Figure 4). In contrast, both CRX-EV and CRX-EEMo treated mice showed preservation of pockets of hematopoietic tissue. While none of the PBS treated or MSC-EV treated mice survived beyond day 20, mice treated with CRX-EVs or CRX-EEMos on day 27 showed significant rebound in BM cellularity. By day 49, there was still maintenance of cellularity. CRX-EV treated mice that received a course of Baytril for *C. bovis* infection survived long-term (day 133) and continued to show robust BM cellularity.

Using a semi-quantitative scoring method for BM cellularity loss, compared to healthy controls, both PBS controls and MSC-EV treated mice at day 6 and 7 post-irradiation showed poor cumulative cellularity scores of 4.8 and 5.0, with severe (<5%-9% hematopoietic tissue present) BM aplasia (Table S3). In contrast, CRX-EV and CRX-EEMo treated mice had slightly better scores of 4.0 and 4.1, corresponding to 20%-29% cellularity. At that time, the mean splenic weight and % spleen BW of both CRX-EV and CRX-EEMo treated mice were comparable to PBS controls. On day 27-29, cellularity scores improved in CRX-EV and CRX-EEMos treated mice, with mean scores of 1.7 ± 0.6 and 2.0 ± 2.6 representing 60%-70% cellularity. By day 49, the BM cellularity continued to remain high in CRX-EVs or CRX-EEMos treated mice. On day 27-29, splenic weights and % spleen BW values in CRX-EV and CRX-EEMos treated mice recovered to levels surpassing healthy controls suggesting induction of extramedullary hematopoiesis. However, at later times points (day 49 or 133), splenic weights and % spleen BW values in both CRX-EV and CRX-EEMos treated mice returned to baseline levels.

### CRX-EEMos have a distinct gene expression profile

Gene expression profiles for relevant cytokines, chemokines and growth factors associated with radioprotection were compared between CRX-EEMos, EEMos and uneducated control monocytes from multiple isolates. Compared to the control monocytes, CRX-EEMos were found to express more than 5000-fold higher levels of IL-6, while increases in IDO-1, FGF2, IL-8, G-CSF, and GM-CSF ranged from 100- to 700-fold higher (Table 1). Other significantly elevated genes (6.7- to 45.0-fold) in CRX-EEMos included IL-10, IL-7, IL-13, MIP-1a, and MIP-1b. When compared to unprimed EEMos, CRX-EEMos showed significantly higher expression of IL-6, IDO, IL-10, IL-7, IL-8, and G-CSF. When comparing the EEMos to control monocytes, a significant increase in expression was detected for IDO, IL-7, IL-8, IL-13, and GM-CSF. Notably the expression level for TNF-alpha, a pro-inflammatory cytokine, for both EEMos and CRX-EEMos remained low and not significantly different from monocyte controls.

**Table 1.** CRX-EEMos show an anti-inflammatory, immunosuppressive, and regenerative gene expression profile.

Gene	EEMos (mean fold increase)	CRX-EEMos (mean fold increase)
**IL-6**	13	5387***$$$
**IDO**	20.8*	792***$
**FGF2**	1.7	298*
**IL-10**	0.8	6.7***$
**IL-7**	1.4**	41.4***$
**IL-8**	4.3**	194***$
**IL-13**	7.1*	15**
**TNF-a**	1.5	1.5
**G-CSF**	3.4	548**$
**GM-CSF**	3.2*	747*
**CCL-5**	1.2	6.4*
**MIP-1a**	1.4	45.4**$
**MIP-1b**	1.3	7.7***$

Multiple isolates of control monocytes, EEMos and CRX-EEMos were collected, RNA isolated and analyzed by qPCR for gene expression. The fold change of gene expression normalized to a GAPDH housekeeping gene was compared to untreated control monocytes. Groups were compared by Kruskal–Wallis with a Dunn post-test.

* *P *≤ .05.

** *P *≤ .005.

*** *P *≤ .0005 were determined as compared to controls or EEMos, designated as ^*^ or $, respectively.

### Gene expression profile of murine monocytes educated with human CRX-EVs shows a species-specific profile

The gene expression profile of primary mouse monocytes educated with human CRX-EVs (mouse CRX-EEMos) showed significantly increased expression of IL-6, IL-10, and CCL-5 (Figure S2, see online supplementary material for a color version of this figure). In contrast to human CRX-EEMos, both the expression of IL-13 and GM-CSF were significantly lower in mouse CRX-EEMos compared to the controls. Interestingly, compared to the human counterpart, gene expression of mouse EEMos showed significantly lower levels for IL-6 (*P *≤ .005) and IL-10 (*P *≤ .05) compared to the mouse monocyte controls.

### CRX-EEMos are enriched for PDL1 but depleted for CD16

**Figure 5.**
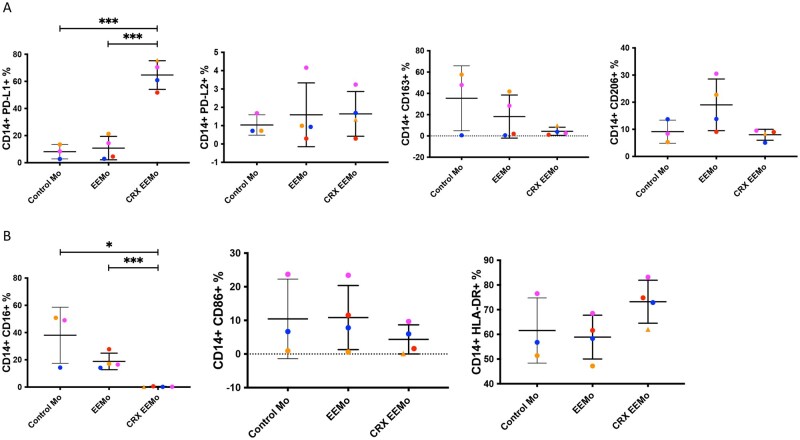
CRX-EEMos shows a significant increase in PD-L1^+^ cells and a significant decrease of CD16^+^ cells. Monocytes from 3 to 4 isolates (each represented in a different color) of either untreated monocytes (control Mo), or monocytes educated with MSC-EVs (EEMos), or CRX-EVs (CRX-EEMos) were analyzed by flow cytometry for (A) M2 markers PDL1, PDL2, CD206, CD163, and CD73 (B) M1 markers CD16, CD86, and HLA-DR. The percentage (%) CD14^+^ cells for each marker (± SE) is shown. The results were pooled from two separate experiments. Groups were compared by Kruskal–Wallis with a Dunn post-test for significance at ^*^P ≤ .05, ^***^P ≤ .0005, between groups.

To determine if CRX-EEMos express cell surface markers consistent with M2 (CD163, CD206, CD73, PD-L1, and PD-L2) or M1 markers (CD16, CD86, and HLA-DR), flow cytometry was performed on CD14^+^ cells from either uneducated control monocytes, EEMos or CRX-EEMos. CRX-EEMos showed a significantly higher percentage of cells with PD-L1 (Figure 5A) and a significantly lower percentage of cells with CD16 (Figure 5B) compared to controls or EEMos. No other M1 or M2 markers were significantly different, suggesting CRX-EEMos are a unique M2-like subset.

### CRX-EEMos release factors that are anti-inflammatory, recruit immune cells, and promote hematopoiesis

**Figure 6.**
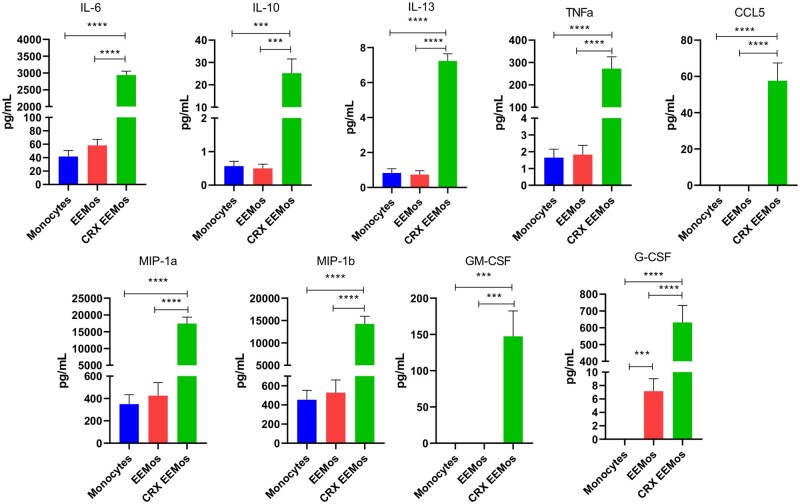
CRX-EEMos secrete high levels of anti-inflammatory cytokines, growth factors and chemokines. Two different sets of MSC-EV and CRX-EV production batches were used to educate three monocyte donor isolates to generate EEMos, CRX-EEMos with untreated control monocytes; for a total of 6 biological replicates for each set. Supernatants collected after 18-24 hours of education and were analyzed in triplicate and compared using Kruskal–Wallis with Dunn’s post-test for significance. ^***^P ≤ .0005, ^****^P ≤ .0001 were determined compared to monocytes, EEMos and CRX-EEMos.

Secretome analysis demonstrated that proteins involved in radioprotection were produced at significantly higher levels by CRX-EEMos. Compared to control monocytes or EEMos, there were significant increases in secreted levels of IL-6 and IL-10 by CRX-EEMos (Figure 6). In addition, CRX-EEMos secreted significantly higher levels of chemokines like CCL-5, MIP-1α, and MIP-1β and growth factors, G-CSF and GM-CSF that stimulate hematopoiesis. While significant increases in TNFα was secreted by CRX-EEMos, other pro-inflammatory cytokines including IL-1β, IL-2, IL-12 (p40/p70), IL-17, and IFNα/IFNγ, were all below the limit of detection (data not shown). The remaining detectable analytes in the multiplex panel; IL-8, IL-1RA, MCP-1, and HGF were all produced at similar levels between groups (Figure S3, see online supplementary material for a color version of this figure). Most of the elevated secreted proteins (IL-6, IL-10, IL-13, CCL-5, MIP-1α, MIP-1β, GM-CSF, and G-CSF) in the CRX-EEMos also showed a respective increase in their gene expression, except for TNFα which showed low expression (Table 1) despite high protein levels. The secretome of EEMos showed a significant increase in G-CSF with modest increases in IL-6, MIP-1α, MIP-1β compared to control monocytes (Figure 6).

### Let-7 antisense oligonucleotides decreased gene expression/protein secretion of IL-10 in CRX-EEMos and increased protein secretion of IL-6 in EEMos

To determine a mechanism by which CRX-EVs regulate monocytes, CRX-EEMos were treated with Let-7 antisense oligonucleotides or nonsense control, then analyzed for their secretome at genomic and proteomic levels as compared to control monocytes and EEMos. Let-7b-5p antisense oligonucleotides treatment decreased IL-10 production in CRX-EEMos, showing decreased expression (*P *= .08; Figure S4A, see online supplementary material for a color version of this figure) and significantly reduced IL-10 protein production (Figure S4B, see online supplementary material for a color version of this figure). This inhibition was specific as no change in IL-10 production was observed in CRX-EEMos treated using either Let-7d-3p antisense oligonucleotides or nonsense controls. In contrast, Let-7d-3p antisense oligonucleotides significantly increased secretion of G-CSF protein without a matching increase in gene expression in CRX-EEMos. In addition, secretion of GM-CSF was nonspecifically inhibited by either the Let-7d-3p antisense or nonsense oligo control. Overall, in monocytes or EEMos treatment with either Let-7 antisense oligonucleotide with showed little change in gene expression or secretion of most proteins when compared to nonsense oligonucleotides (data not shown). However, treatment of EEMos with either Let-7 antisense inhibitor generated a significant (*P *≤ .0001) and specific increase in secreted IL-6 without significant change in gene expression (Figure S4C and D, see online supplementary material for a color version of this figure), indicating that both Let-7 miRNAs regulate IL-6 production by EEMos.

## Discussion

H-ARS is a life-threatening consequence of exposure to radiation that can occur either from radiopharmaceuticals or due to accidental or intentional exposure to nuclear radiation. Based on their polarization state, macrophages play a key role in treating H-ARS.^29^ While current FDA-approved drugs like GM-CSF stimulate macrophage expansion, next generation therapies are needed to promote their innate ability to attenuate inflammation, accelerate hematopoietic reconstitution and promote tissue remodeling.^30-32^ Here we show that EVs from CRX-primed human MSCs used to educate monocytes *in vivo* or *ex vivo* induces long-term survival in a xenogeneic H-ARS mouse model by polarizing monocytes into a M2-like PD-L1+ phenotype capable of producing cytokines (IL-6, IL-10), chemokines (CCL-5, MIP-1α, MIP-1β), and growth factors (G-CSF, GM-CSF) that induce hematopoiesis, improve recovery of CBCs and BM cellularity. Moreover, enriched Let7 miRNAs in CRX-EVs appear to be involved in IL-10 and G-CSF production.

We previously demonstrated the role of TLR4 in driving radio-mitigator properties in macrophages using LPS.^19^ While LPS is a potent TLR4 agonist, its toxicity from even low levels of endotoxin within EV preparations may potentially induce septic shock. Moreover, even clinical grades of LPS can contain bacterial nucleic acids known to bind other TLRs groups that can potentially elicit unintended inflammatory symptoms.^33^ CRX-527, a synthetic lipid A mimetic without an endotoxin domain, has a safety profile expected to be similar to many adjuvants currently used in human vaccines.^22^^,^^34^^,^^35^ Its potency is also high, requiring at least 10-fold less to get similar effects as LPS for monocytes; likely due to its increased purity and ability to activate TLR-4 without CD14 co-receptor binding.^36^ While we found that treatment with CRX-527 alone was ineffective in our H-ARS model, it is plausible that the effectiveness of free CRX-527 may be augmented when bound to EVs secreted by MSCs stimulated via TLR-4.

Examining the identity of CRX-EVs, nine core surface markers were present out of 37 tested, with a significant increase in CD44, a receptor for hyaluronic acid, (HA). Since CD44 is constitutively expressed on MSCs, CRX-527 binding may transcriptionally upregulate CD44, thus, enriching its incorporation onto its secreted CRX-EVs. Since HA plays a key role in wound healing, future studies may need to determine if CRX-EVs assist with binding of cells involved in tissue repair to sites of tissue damage.^37^ Macrophages also produce more HA in response to tissue injury, so the increased expression of CD44 in CRX-EVs may target them to macrophages at repair sites.^38^ Moreover, MSC-EVs treated with high molecular weight HA increases uptake by monocytes and enhance trafficking to sites of inflammation.^39^

After education by CRX-EVs, the CRX-EEMos produce IL-6, whose importance was previously shown with LPS-EEMos.^20^ High levels of anti-inflammatory cytokines, like IL-10 and IL-13, were also released by CRX-EEMos.^40^ G-CSF and GM-CSF, both FDA approved therapies to treat ARS, were also secreted in high levels by CRX-EEMos. Significant levels of several chemokines including CCL-5, MIP-1α and MIP-1β, were also produced by CRX-EEMos. While their primary function is to attract and promote immune cell infiltration to inflamed tissue sites, they have been reported to work synergistically to inhibit the release of the proinflammatory cytokine IL-1β,^41^^,^^42^ which can stimulate a systemic inflammatory response syndrome (SIRS), a serious complication of ARS.^43^ Indeed, undetectable levels of IL-1β were found in CRX-EEMos. Furthermore, except for TNFα, the secretion of other pro-inflammatory cytokines (IL-2, IL-12 (p40/p70), IL-17, IFNα, IFNγ) known to worsen damage from radiation exposure were undetectable in CRX-EEMos.^44^ Lastly, enrichment for PD-L1-expressing CRX-EEMos could inhibit Th1 cell function shifting to Th2 activity promoting immune tolerance.^45^

Enriched levels of Let-7 family miRNAs were previously identified from two independent RNA-seq studies analyzing both MSC-EVs and CRX-EVs.^21^^,^^23^ Antisense inhibitors against two miRNA let-7 subtypes (let-7d and 7b) were tested to determine any downstream effects on CRX-EV education of monocytes. In humans, subtypes of the Let-7 family (a-g and i) share a high degree of sequence similarity and are primarily known as tumor suppressors that downregulate the expression of target genes such as MYC, KRAS, and LIN-28 effecting cell differentiation, growth and survival.^46^ In macrophages, Let-7 miRNAs can promote M2 polarization and stimulate the production of cytokines such as IL-6 and IL-10 through the NF-kappa B pathway.^47-49^ Let-7 members can also modulate TLR-4 signaling in cells leading to the tolerization of stimuli such as LPS.^50^ We found Let-7 miRNA antisense inhibition of CRX-EV educated monocytes did indeed impact IL-10, G-CSF and to a lesser extent IL-6, production. IL-10 is reported to be radioprotective, mediating hematopoietic recovery by suppressing both pro-inflammatory cytokines and radiation-induced apoptosis of BM stem cells.^51-53^ Other enriched miRNAs found in the CRX-EVs; including miR-34a, miR-26a, miR-181a, and miR-455a known to promote M2 macrophage polarization and modulate inflammatory responses should be investigated in future studies.^23^^,^^54^

Overall, the use of CRX-EVs warrants further investigation as a therapy for H-ARS. A practical radiotherapeutic should be easily stored, available in adequate quantities and ready for use within hours after exposure. EVs can retain the ability to remain therapeutically stable when stored frozen long-term and can be available immediately upon thaw.^55^ For sufficient doses after a mass casualty incident, EVs from MSCs made using GMP-compliant biomanufacturing platforms such as Cellstack or bioreactors are actively being developed.^21^^,^^56-58^ However, producing clinical grade-EVs for ARS under GMP requires finely tuned upstream processes (MSC source selection, type of scale-up platform) and downstream purification/characterization workflows that balance yield, purity and consistent bioactivity. When closer to a scale-up procedure, methods to quantitate any carryover of CRX-527 in the CRX-EVs preparation using a combination of cell-based TLR-4 assays coupled with proteomic quantitation of CRX-527 in EVs should be planned. While the literature supports the relevance of using human MSCs/EVs in murine models of H-ARS^20^^,^^59-62^, validation of CRX-EVs and CRX-EEMos will be needed in immuno-competent syngeneic murine models and humanized models to understand species-specific immune interactions and receptor compatibility. Studies of CRX-EVs in immunocompetent mice at higher radiation doses, CRX-EEMos in humanized mice and biodistribution studies by positron emission tomography are also underway.^63^ Ultimately, testing in large animal models of H-ARS will be needed to verify potency observed in rodent models and satisfy the FDA Two-Animal Rule for approval.

## Conclusion

A single *in vivo* infusion of EVs from human MSCs primed with the synthetic TLR4 agonist CRX-527 provides long-term survival in lethal H-ARS models by promoting tissue repair in the BM, inducing hematopoiesis in the BM and spleen and promoting hematopoietic recovery through restoration of normal CBCs. CRX-EVs can also be used *ex vivo* to educate human monocytes to generate CRX-EEMos to treat H-ARS. Mechanistically, we believe that M2-like macrophages generated endogenously or *ex vivo* by CRX-EVs secrete anti-inflammatory and pro-repair factors that preserve stromal and endothelial cells in the irradiated marrow niche. They also help deposit extracellular matrix proteins and release pro-growth cytokines that recruit, support and expand surviving hematopoietic stem and progenitor cells. Overall, the direct use of CRX-EVs to treat H-ARS is an attractive option to treat exposures to ionizing radiation.

## Supplementary Material

szaf068_Supplementary_Data

## Data Availability

The datasets used and/or analyzed during the current study are available from the corresponding author on reasonable request.
