# Supplementary material for: Extracellular vesicles from mesenchymal stromal cells primed with synthetic toll-like receptor 4 agonists treat hematopoietic acute radiation syndrome
Source: Stem Cells Transl Med. 2026 Jan 9;15(1):szaf068. doi: 10.1093/stcltm/szaf068 (PMC12784198; doi:10.1093/stcltm/szaf068)
Supplement: szaf068_Supplementary_Data [file szaf068_supplementary_data.zip › 12-Dec-2025_063415_Supplemental_Methods_and_Data.pdf]

Supplementary Information

Extracellular vesicles from mesenchymal stromal cells primed with synthetic toll-like receptor 4 agonists treat hematopoietic acute radiation syndrome

John A. Kink, PhD[1-3], Matthew H. Forsberg, PhD[3], Derek M. Krismer[1], Anna S. Thickens[1], Raghavan Chinnadurai, PhD[4], Alex S. Chen[1,3], Daniel J. Chacko[1], Melissa Graham, DVM[5], Peiman Hematti, MD[6], Christian M. Capitini, MD[2,3]

[1]Department of Medicine, University of Wisconsin School of Medicine and Public Health, Madison, Wisconsin, 53705.

[2]University of Wisconsin Carbone Cancer Center, Madison, Wisconsin, 53705.

[3]Department of Pediatrics, University of Wisconsin School of Medicine and Public Health, Madison, Wisconsin, 53705.

[4]Department of Biomedical Sciences, Mercer University School of Medicine, Savannah, GA, 31404.

[5]The Comparative Pathology Laboratory, Research Animal Resource Center, University of Wisconsin, Madison, Wisconsin, 53705.

[6] Division of Hematology/Oncology, Medical College of Wisconsin, Milwaukee, Wisconsin, 53226.

* Corresponding authors: Christian Capitini, 1111 Highland Ave, WIMR 4137, Madison, WI 53705, email: ccapitini@pediatrics.wisc.edu; Peiman Hematti, 9200 W. Wisconsin Ave, Milwaukee, WI 53226 email: hematti@mcw.edu

Running title: CRX-primed MSC-EVs for ARS

Conflict of interest statement: J.A.K, P.H. and C.M.C. are inventors on patents related to this publication (US Patents 10,166,254 and 11,499,730). C.M.C. reports honorarium from Bayer and Novartis, as well as equity interest in Elephas, who had no input in the study design, analysis, manuscript preparation, or decision to submit for publication. No other relevant conflicts of interest are reported.

Keywords: extracellular vesicles; exosomes; mesenchymal stromal cells; TLR4; hematopoietic acute radiation syndrome; monocytes; radiomitigator.

Supplementary Information

Supplemental Methods

Isolation of EVs from human MSCs

BM-MSCs were grown in alpha MEM media (Corning CellGro, Manassas, VA) supplemented with 10% heat-inactivated fetal bovine serum (FBS) (Hyclone, Logan, UT), 100X l-Ala-l-Glutamine (Corning GlutaGro), and 100X NEAA (Corning) in T75 flasks. MSCs were then incubated for with or without CRX-527 for 18–24 hours and the conditioned SFM was collected and processed to isolate the EVs. The conditioned SFM containing the EVs was centrifuged at a low-speed spin (2000 x*g* at 4°C for 20 minutes) to remove any cell debris and the supernatant was then ultra-centrifuged for 2 hours using Optima™ L-80XP Ultracentrifuge (Beckman Coulter Inc, Brea CA, USA, USA) at 100,000 xg_{avg} at 4°C. The pellet containing EVs was resuspended in PBS based on the initial SFM volume and stored at -80°C.

Surface marker analysis of EVs by flow cytometry

Characterization of EVs was performed by flow cytometry using the MACSPlex Exosome Kit (Miltenyi Biotec, Bergisch Gladbach, Germany) according to the manufacturer's protocol. EVs were mixed with an equal volume of capture beads coupled with exosome surface marker antibodies plus two isotype controls and gently rotated in the dark at 4 °C overnight. The bead-exosome complexes were washed and then incubated for 1 hour with a detection bead mixture consisting of pan-exosome markers CD9, CD63, and CD81 labeled with FITC, PE, or APC. The beads were then washed and resuspended in MACSPlex buffer for analysis. Prior to experimentation, the system was calibrated, and background settings were adjusted to unlabeled beads. Batch analysis quantified median intensities for each bead population and analyte surface expression was calculated for each sample. A Miltenyi MACSQuant Analyzer 10 was utilized for sample acquisition and MACSQuantify Software v. 2.13 was used for data analysis. Median fluorescent values of 1.0 or more were considered positive and means from EVs isolated from different MSC isolates were determined.

Western blot analysis of EVs

Western blot analysis was performed using a Western blot sampler kit (SBI; catalog# EXOAB-KIT-1) for detection of the cytoplasmic marker Hsp70. Briefly approximately 50ug of each sample was prepared and proteins separated on a SDS polyacrylamide gel. Proteins were transferred to PVDF membrane and probed with specific antibodies according to manufacturer's instruction.

Education of monocytes by EVs

Isolated monocytes were cryopreserved in the vapor phase of liquid nitrogen before use. Monocytes were thawed and cultivated in Iscove's modified Dulbecco's media (Gibco Life Technologies, Grand Island, NY USA) supplemented with 10% human AB serum (Valley Biomedical, Winchester, VA, USA), 1× MEM nonessential amino acids (Mediatech, Manssas, VA, USA), 1x Glutagro (Mediatech), 1x sodium pyruvate (Mediatech), and 4 ug/mL human recombinant insulin (Life Technologies, Grand Island, NY, USA). Monocytes at 10^{-7} in 10 ml of culture media were educated with $5x10^9$ MSC-EVs or CRX-EVs or uneducated (controls) for 18-24 hours as previously described.[20] After incubation, control monocytes, EEMos or CRX-EEMos were collected by centrifugation and washed with PBS at 300 xg for 10 minutes at 4°C.

Clinical scoring system for the H-ARS model

Mice were typically monitored at least 3 times per week with clinical scores and survival recorded for each mouse. Clinical scores were determined based on five clinical categories on a modified clinical scoring system with a score from 0 to 2 (normal to poor) and a cumulative score was determined for each mouse. Specifically, these categories are as follows: *Weight loss*: grade 0 < 10%, grade 1 > 10 to < 25%, and grade 2 > 25%; *Posture*: grade 0 normal, grade 1 hunching noted at rest, and grade 2 severe hunching impairs movement; *Activity*, grade 0 normal, grade 1 mild to moderately decreased, grade 2 stationary unless stimulated; *Fur texture*: grade 0 normal, grade 1 mild to moderate

ruffling, grade 2 severe ruffling or poor grooming; and *Skin Integrity*: grade 0 normal, grade 1 scaling of paws or tail, and grade 2 obvious areas of the exposed skin.

Histological preparation of the BM in the ARS model

Long bones were fixed with 10% neutral buffered formalin and a separate approximately 40-minute immersion in Surgipath Decalcifier I (Leica, USA). Samples were processed on a Tissue-Tek VIP 6 processor (Sakura Finetek, Torrance, CA) and embedded on a Tissue-Tek embedding station (Sakura). Slides were cut on a Leica 2235 microtome at 5-6 microns and stained with H&E using a Tissue-Tek DRS 2000 automatic stainer (Sakura). Tissues were visualized using a Nikon Eclipse 50i microscope at multiple magnifications using objectives: 4x/0.10 Plan, 10x/0.25 Plan, 20x/0.40 Plan, 40x/0.65 Plan. Photographs were taken using a Swiftcam 18-megapixel camera aided with acquisition software for Mac (Swift Imaging 3.0) at 400x total magnification.

Gene expression analysis of EV educated human or mouse monocytes

RNA from 3-4 human isolates of uneducated monocyte controls or 10^{-7} monocytes educated with $5x10^{9}$ MSC-EVs or CRX-EVs was isolated using a RNeasy micro kit, (Qiagen, Valencia, CA, USA) and quantitated with A260/A280 using an Epoch microplate reader (BioTeK Instrument Inc, Winooski, VT, USA). The isolated RNA was converted to cDNA using Verso cDNA synthesis kit (Thermo Scientific, Pittsburgh, PA, USA) and a Veriti Thermal Cycler (Applied Biosystems, Foster City, CA, USA). Quantitative polymerase chain reaction (qPCR) was performed using Power SYBR green master mix (Applied Biosystems) on StepOne Plus instrument (Applied Biosystems) using standard protocols. The comparative threshold cycle method (Ct) was used to calculate the mRNA levels. Ct values for the *GAPDH* housekeeping gene and the genes of interest were determined, and the difference between the Ct values of each gene of interest and the mean *GAPDH* Ct was calculated (delta Ct). Differences in the delta Ct (delta-delta Ct) of genes in EEMos and CRX- EEMos were normalized to monocyte controls. Reverse transcriptase –PCR data is presented as fold change expression=$2^{-delta,delta\ Ct}$ of each gene in comparison with the monocyte controls.

For mouse monocyte gene expression, primary mouse monocytes from C57BL/6 mice (Cat # 1117 Cellero, Northridge, CA) were either uneducated or 10^{-7} cells educated with $5x10^{9}$ human MSC-EVs or CRX-EVs in DMEM with 10% FBS. Isolation of RNA, cDNA and qPCR analysis was performed as described above using mouse *GAPDH* housekeeping gene and the genes of interest.

Flow Cytometry of EV-educated human monocytes

Monocyte from multiple donors (3-4) of controls (uneducated), EEMos and CRX-EEMos, were harvested, counted with a Beckman Coulter Z1 Particle Counter and then $1x10^{6}$ cells were incubated with Fc block (BD Pharmingen, San Jose, CA, USA, cat#: 564220) for 10 minutes at room temperature. Cells were then stained at 4°C for 20-30 minutes with anti-human antibodies in staining buffer (PBS with 2% FBS). Antibodies were purchased from BioLegend (San Diego, CA) and included: CD206: (15-2, cat# 321105), CD163: (GHI/61, cat# 333617), PD-L1: (29E.2A3, cat# 329721), PD-L2: (24F.10C12, cat# 329608), CD14: (HCD14, cat# 325627), CD16: (3G8, cat# 302025), HLA-DR: (L243, cat# 307639), CD73: (TY/11.8, cat# 127223), and CD86: (IT2.2, cat# 305431). The cells were washed with PBS, centrifuged at 300 xg for 10 minutes, and Ghost Dye™ Red 780 viability dye (cat# 13-0865, Tonbo Biosciences, San Diego, CA) was added for 20 minutes at room temperature. Cells were then washed with staining buffer, spun down, and resuspended in staining buffer. Cells were then run on an Attune™ NXT flow cytometer (Thermo Fisher Scientific). Subsequent analysis was performed using Flowjo™ software (Becton Dickinson, Franklin Lakes, NJ).

Let-7 miRNA inhibition studies in EV educated monocytes

Primary human control monocytes, EEMos and CRX-EEMos were each either uninhibited or treated with miRNA antisense inhibitors to Let-7b, Let-7d or nonsense control and compared for changes in gene expression by qPCR and protein secretion by multiplex ELISA. Specifically, monocytes in six well plates were either untreated (controls) or treated with MSC-EVs or CRX-EVs as described, then incubated for 4 hours to allow for uptake and education. All cells

were then treated with transfection reagent (HiPerfect Qiagen) facilitate anti-sense miRNA uptake with the inhibitor sets also receiving either Let-7b-5p (miRCURY LNA miRNA power inhibitor, 339131 YI04100945-DDA, Qiagen), Let-7d-3p (miRCURY LNA miRNA power inhibitor, 339131 YI04102463-DDA, Qiagen) or a negative control nonsense oligonucleotide (miRCURY LNA miRNA power inhibitor control, 339131 YI00199006-DDA) at a concentration recommended by the manufacturer based on cell number. After incubation for 48 hours, the cells and supernatants were harvested by centrifugation. RNA was isolated and quantitated followed by cDNA synthesis as described. Gene expression analysis for IL-6, IL-10, IDO-1, FGF2, G-CGF, GM-CSF was performed by qPCR as described. Cell culture supernatants were analyzed secreted proteins for IL-6, IL-10, IDO-1, G-CGF, GM-CSF, IL-13, TNF-alpha, MIP-1 alpha, MIP-1 beta, and FMS-like tyrosine kinase 3 ligand (FTL3L) using a custom multiplex panel (Meso Scale Diagnostics, Rockville MD).

Supplementary Information

Supplemental Table and Figure Legends

Supplemental Table 1. CBCs of mice treated with CRX-EVs administered 24 hours after lethal radiation. Mice were treated with vehicle (PBS), MSC-EVs and CRX-EVs and bled for complete blood counts (CBCs) to determine the effects of radiation in mice by comparing blood pre-radiation (pre-rad controls) with blood post-radiation collected at strategic time points: early post-challenge on (day 7), at early recovery period, (days 19-20) and during the late recovery period (days 43 and 75) then assayed using a Hemavet 950FS analyzer (Drew Scientific Inc., Miami Lakes, FL). CBC means were determined from at least 3 mice/group and groups and compared by principal component analysis and *t* tests.(pre-rad) controls. n/a = not applicable. * $p \leq 0.05$, ** $p \leq 0.01$, ***$p \leq 0.005$.

Supplemental Table 2. CBCs of mice treated with CRX-EEMos administered 24 hours after lethal radiation. Mice were treated with vehicle control (PBS), EEMos, or CRX-EEMos and bled for complete blood counts (CBC) to determine the effects of radiation in mice by comparing blood pre-radiation (pre-rad controls) with blood post-radiation collected at strategic time points: early post-challenge on (day 5-6), and at a mid-recovery period, (days 29-30) then assayed using a Hemavet 950FS analyzer (Drew Scientific Inc., Miami Lakes, FL). Means were determined from at least 3 mice/group and groups were compared by principal component analysis and *t* tests. n/a= not applicable. *$p \leq 0.05$, **$p \leq 0.01$, ***$p \leq 0.005$ as compared to pre-radiation (pre-rad) controls. For EEMos #, only one surviving mouse; statistical comparison was not possible.

Supplemental Table 3. Mice treated with CRX-EV or CRX-EEMos after lethal radiation showed improved bone marrow cellularity. Bone marrow cellularity scores and spleen weights were determined in mice treated with CRX-EV or CRX-EEMos after lethal radiation. Data presented on different days (6-7, 27-29, and 49) post-radiation. Day 133 represents data from mice treated with CRX-EVs at 24 hours post-radiation. The scoring of mean cellularity loss in the BM of femurs were graded from 0 to 5 (indicating most to least amount of cellularity) as a percent expressed as: 0.0 = 90%-100% (minimal or no BM loss), 1.0 = 80%-89%, 1.5 = 70%-79%, 2.0 = 60%-69%, 2.5 = 50%-59%, 3.0 = 40%-49%, 3.5 = 30%-39%, 4.0 = 20%-29%, 4.5 = 10%-19%, and 5.0 = <5%-9% (severe BM loss). The mean spleen weights and spleen weights as percentage (%) of total body weight (BW) are also shown. Data were generated from 3 to 4 mice/group and compared by Kruskal–Wallis with a Dunn post-test compared to the PBS control. *$p \leq 0.05$

Supplemental Figure 1: CRX-527 alone administered 4 hours after lethal radiation was not significantly effective against H-ARS in xenogeneic mouse model. On day 0, NSG mice received 4 Gy of lethal radiation followed by an i.v. treatment administered 4 hours later with vehicle (PBS), or CRX-527 at 1ug/100 ul of PBS. **(A)** Survival curve of irradiated mice treated 4 hours after radiation. **(B)** Mean % weight loss **(C)** Mean clinical score (collective score of percent weight loss, posture, activity, and fur texture). Results were evaluated using Log-rank (Mantel Cox) to compare survival curves and Kruskal-Wallis with Dunn's post-test was used for comparing % weight change and mean clinical scores. Results of **A-C** are from one experiment, with 5 to 8 mice/group.

Supplemental Figure 2: **Murine monocytes educated with human CRX-EVs show increased gene expression for IL-6, IL-10 and CCL-5.** Primary mouse monocytes were either untreated (control mouse monocytes), treated for 24 h with human MSC-EVs or CRX-EVs to produce murine EEMos, or murine CRX-EEMos. After treatment, the cells were collected and RNA isolated and analyzed by q-PCR for gene expression. The fold change of gene expression normalized to a GAPDH housekeeping gene was compared to untreated control monocytes. Results are from replicates of two independent studies. Groups were compared by Kruskal-Wallis with a Dunn post-test. *$p \leq 0.05$, **$p \leq 0.005$, and *** $p \leq 0.0005$ between groups are designated as compared to control monocytes.

Supplemental Figure 3. IL-8, IL-1RA, MCP-1, and HGF are secreted at similar levels by CRX-EEMos EEMos and control monocytes. Three monocyte isolates were educated for 24 hours with MSC-EVs (EEMos) or CRX-EVs (CRX-EEMos) from two separate EV production batches and compared to untreated monocytes. Each set was run in triplicate and after 24

hours supernatants were assayed using a cytokine magnetic 30-Plex panel Luminex kit. Media with and without EVs were run as controls. Groups were compared by Kruskal-Wallis with a Dunn post-test.

Supplemental Figure 4. **Let-7 antisense oligonucleotides decreased gene expression and protein secretion of IL-10 in CRX-EEMos and increased protein secretion of IL-6 in EEMos** . Untreated CRX-EEMos controls or CRX-EEMos treated with oligonucleotide inhibitors (nonsense, Let-7b or Let-7d antisense) were analyzed for changes in gene expression in cells by q-PCR and secreted proteins in culture supernatants by multiplex ELISA. The Let 7 antisense oligo treated CRX-EEMos showed changes in gene expression and secretion of G-CSF, GM-CSF and IL-10. **(A)** Comparison of fold change in gene expression between untreated and treated CRX-EEMos first normalized to a GAPDH housekeeping gene compared to untreated monocytes. (**B)** Comparison of secreted protein between untreated and treated CRX-EEMos detected in picogram/ml. (**C)** The treatment with Let 7 antisense oligos also showed changes in secretion of IL-6 in EEMos. Comparison of fold change in gene expression of IL-6 between untreated and treated EEMos were first normalized to a GAPDH housekeeping gene compared to untreated monocytes. (**D)** Comparison of secreted IL-6 protein between untreated and treated EEMos detected in picogram/ml. Significance between the antisense oligonucleotide treated groups were compared by Kruskal-Wallis with a Dunn post-test between inhibited CRX-EEMos or EEMos as compared to their respective untreated controls. **** $p \leq 0.0005$.

Supplemental Figure 1

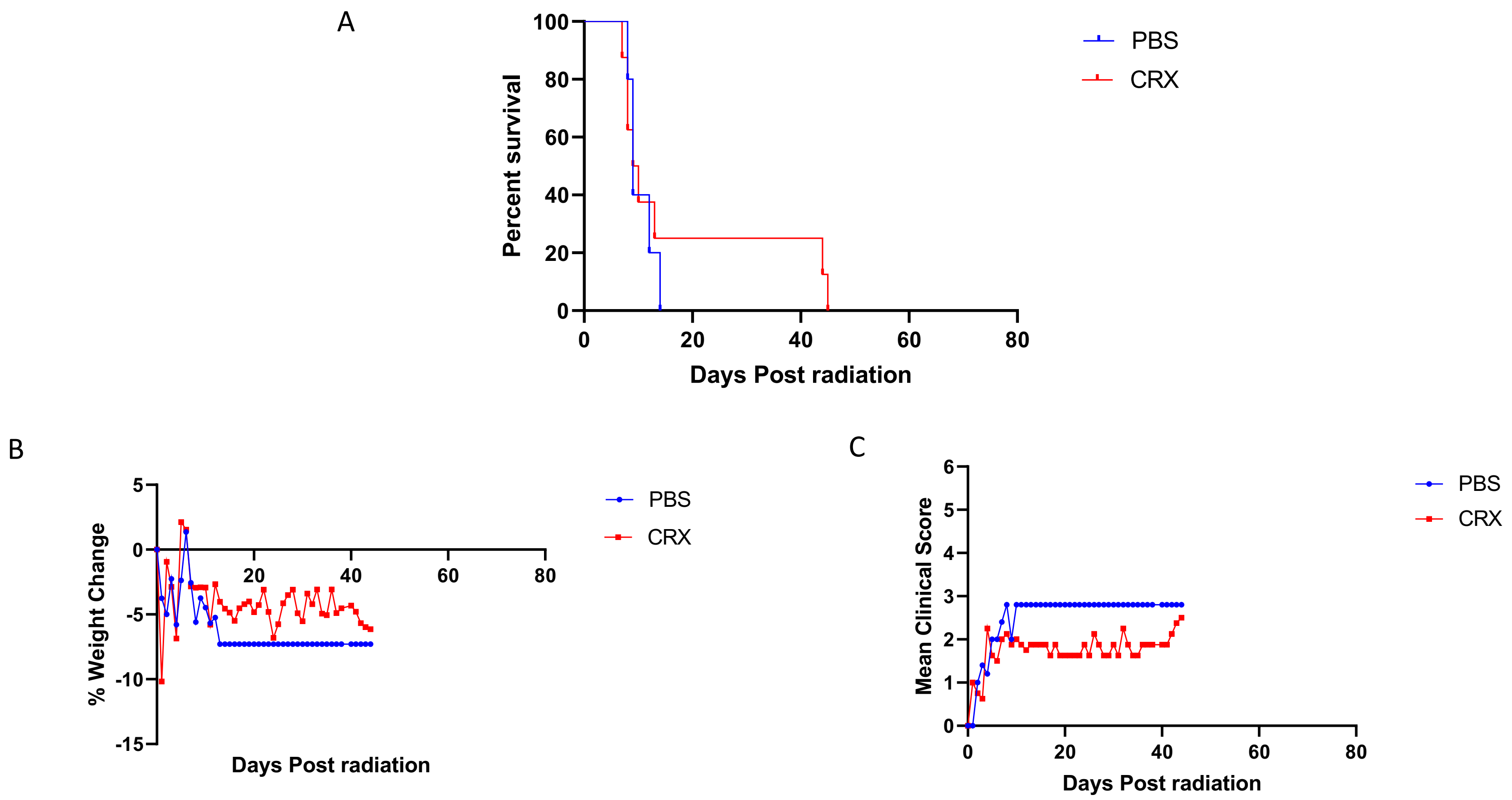

Supplemental Figure 2

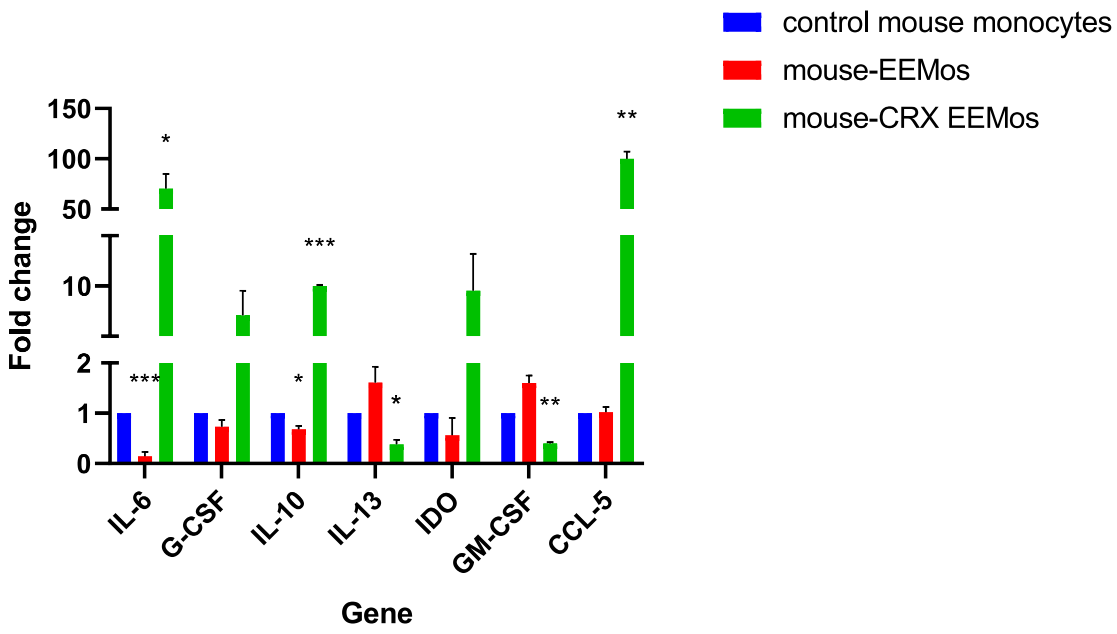

Supplemental Figure 3

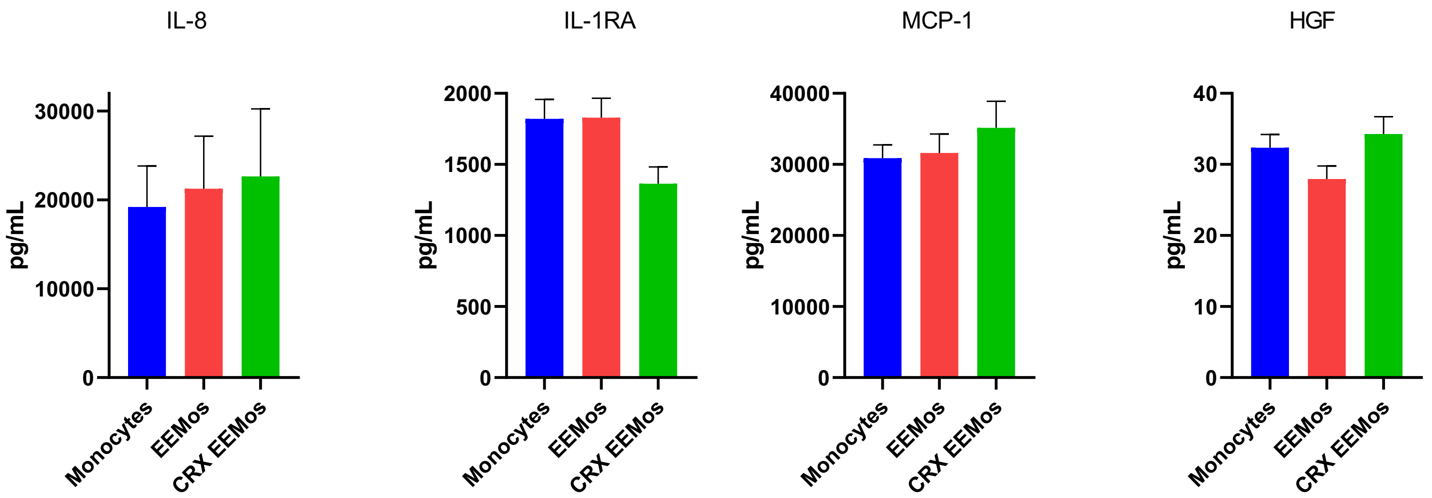

Supplement Figure 4

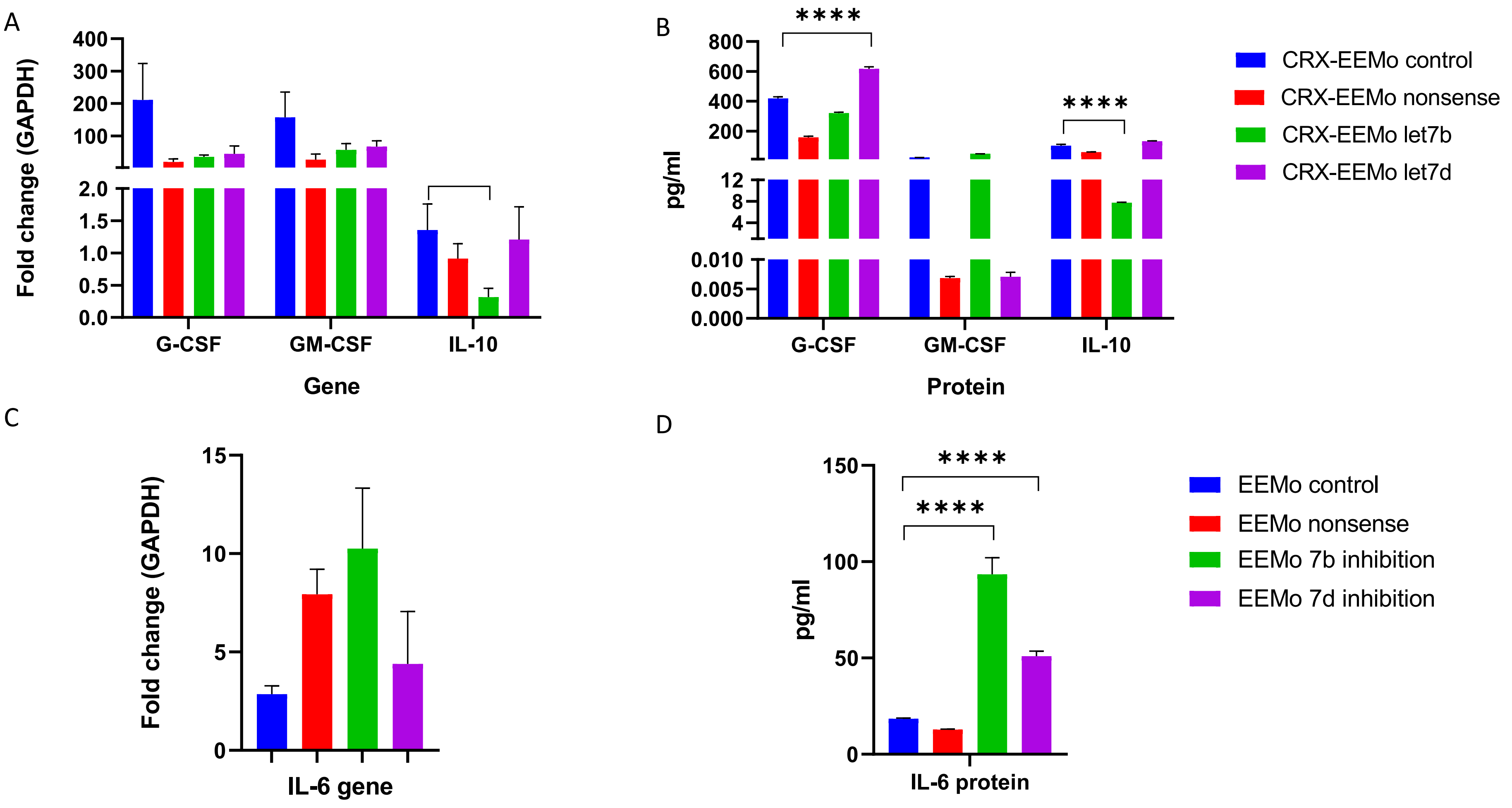

Supplemental Table 1

Group	***Days-post rad***	***RBC (M/ul)***	***WBC (K/ul)***	***Neutrophils (K/ul)***	***Lymphocytes (K/ul)***	***Monocytes (K/ul)***	***Platelets (K/ul)***	***Platelet volume (fL)***
Pre-rad Control	n/a	7.3	2.2	1.2	0.65	0.22	790	4.9
PBS	7	6.4	0.24*	0.08*	0.1	0.05*	85***	5.6*
MSC-EVs	7	7.7	0.24*	0.05*	0.05	0.05*	99***	6.1***
CRX-EVs	7	6.4	0.41*	0.12*	0.14	0.05*	95***	6.4***
PBS	19	3.42	0.42	0.15	0.16	0.09	115	6.1
MSC-EVs	20	2.95	0.24	0.01	0.01	0.01	64	6.4
CRX-EVs	20	5.8	0.85	0.29*	0.47	0.05	355*	5.9*
MSC-EVs	43	6.2	0.98	0.62	0.26	0.08	199	6.8
CRX-EVs	43	7.9	2.4	1.7	0.53	0.16	538	5.4
CRX-EVs	75	6.3	1.6	0.8	0.43	0.3	522	5.0

Supplemental Table 2

Group	*Day post radiation*	*RBC (M/ul)*	*WBC (K/ul)*	*Neutrophils (K/ul)*	*Lymphocytes (K/ul)*	*Monocytes (K/ul)*	*Platelets (K/ul)*	*Platelet volume (fL)*
Pre-rad control	n/a	9.0	2.6	1.4	0.8	0.2	1019	4.9
PBS	5-6	8.5	0.19***	0.01*	0.08*	0.02***	281***	4.8
EEMos	5-6	9.4	0.2*	0.05*	0.05	0.02*	342***	4.9
CRX- EEMos	5-6	7.1	0.24**	0.03**	0.07*	0.01**	179***	5.4*
EEMos[#]	29-30	4.7	0.38	0.17	0.15	0.05	247	6.8
CRX- EEMos	29-30	7.0	1.2	0.8	0.23	0.12	310***	6.1***

Supplemental Table 3

Group	***Day post-radiation***	***Mean Cellularity score (femur)***	***Mean Spleen weight (mg)***	***Mean % spleen BW***
Healthy controls	N/A	0.0 +/- 0.0	29.2 +/-3.1	0.12 +/-0.02
PBS controls	6-7	4.8 +/- 0.3	9.3 +/-3.8	0.05 +/-0.01
MSC-EVs	6-7	5.0 +/-0.0	5.8 +/-1.2	0.04 +/- 0.004
CRX-EVs	6-7	4.0 +/- 0.5	8.6 +/- 1.2	0.04 +/-0.004
CRX-EEMos	6-7	4.1 +/- 0.3	10.3 +/- 0.6	0.05 +/- 0.002
CRX-EVs	27-29	1.7 +/- 0.6*	62.6 +/- 39.7	0.36 +/- 0.25
CRX-EEMos	27-29	2.0 +/- 2.6	80.6 +/- 50.7	0.45 +/- 0.28
CRX-EVs	49	1.7 +/- 0.6*	29.3 +/- 16.7	0.15 +/- 0.06
CRX-EEMos	49	1.5 +/- 0.5*	22.2 +/- 14.9	0.12 +/- 0.7
CRX-EVs	133	1.6 +/- 1.5	N/A	N/A
