# Supplementary material for: Extracellular vesicles from mesenchymal stromal cells primed with synthetic toll-like receptor 4 agonists treat hematopoietic acute radiation syndrome
Source: Stem Cells Transl Med. 2026 Jan 9;15(1):szaf068. doi: 10.1093/stcltm/szaf068 (PMC12784198; doi:10.1093/stcltm/szaf068)
Supplement: szaf068_Supplementary_Data [file szaf068_supplementary_data.zip › Supplementary_Data.zip!/STCLTM Financial Disclosure Form (1).pdf]

STEM CELLS Translational Medicine
POTENTIAL CONFLICT OF INTEREST AND FINANCIAL DISCLOSURE FORM

Manuscript Title: Extracellular vesicles from mesenchymal stromal cells primed with synthetic toll-like receptor 4 agonists treat hematopoietic acute radiation syndrome

Name of Discloser: Melissa Graham

I am a: ☐ Corresponding Author ☒ Co-Author

It is the policy of **STEM CELLS Translational Medicine**® Journal to ensure balance, independence, objectivity, and scientific rigor in all of its educational activities through the disclosure of financial interests and other relationships.

POLICY: In accordance with Journal policy, all persons who affect the content of an article regarding the products or services of a commercial interest must disclose any financial relationships with that commercial interest occurring within the last 24 months. **STEM CELLS Translational Medicine** abides by a policy of anonymous peer review: any potential conflicts of interest are reviewed by the Editorial Board with the ad hoc assistance of external reviewers and are resolved prior to publication.

DEFINING A CONFLICT OF INTEREST: A conflict of interest exists when an individual has both a financial relationship with a commercial interest and the opportunity to affect the content of an article about the product or services of that commercial interest.

DEFINING A COMMERCIAL INTEREST: A commercial interest is defined as any entity producing, marketing, re-selling, or distributing health care goods or services consumed by, or used on, patients.

STEM CELLS Translational Medicine has faith in the integrity of the individuals who present educational activities. However, to avoid the appearance of any conflict of interest, this form has been adopted to identify and resolve any potential conflicts of interest.

- Relationships with governmental agencies (e.g., the National Institutes of Health) do not have to be disclosed.
- Principal investigators must report research funding relationships under "Research" even if those funds were disbursed to an institution. In addition to your own disclosure, you must disclose financial relationships your spouse or life partner has with applicable commercial interests.
- In addition to your own disclosure, you must disclose financial relationships your spouse or life partner has with applicable commercial interests.

You may wish to double check your disclosure against other sources such as the Open Payments database to ensure that any publicly reported financial relationships have not been omitted in your response. Failure to provide complete and accurate disclosure violates Journal policy and may result in errata, retraction, or other consequences. Authors are solely responsible for ensuring that their disclosures are accurate. (https://openpaymentsdata.cms.gov/)

Do you have any financial relationships to report?

☒ **Yes** ☐ **No**

If yes, please provide relevant information in table below.

Types of financial relationships and the companies with whom we (I and/or my spouse/life partner) have relationships are as follows:

Check boxes	Type of financial relationship WITHIN THE PAST 24 MONTHS ONLY	Indicate applicable commercial interest(s) WITHIN THE PAST 24 MONTHS ONLY	Compensated ✓	Uncompensated ✓
☒	Employment/leadership position	Melissa Graham and spouse both employed by Labcorp	☒	☐
☐	Intellectual property rights/Inventor or patent holder		☐	☐
☐	Consultant/Advisory role		☐	☐
☐	Honoraria received directly from commercial interest or their agents (e.g., speakers' bureaus)		☐	☐
☐	Research funding/Contracted research (including funds paid to your institution)		☐	☐
☐	Ownership interest (stocks, stock options, or other ownership interest excluding diversified mutual funds)		☐	☐
☐	Expert testimony		☐	☐
☐	Other (including any reimbursed or sponsored travel)		☐	☐

If you reported relationships in the chart above, will you nonetheless be able to provide an unbiased article? ☒ **Yes** ☐ **No**

By signing below, I represent that the foregoing information is complete and truthful. I will notify the editorial office if there are any changes in my financial relationships prior to the publication of this article.

Melissa Graham (signature)

Signature of Reporting Individual

05Nov2025

Date of Submission
